# Metabolism of Mycosporine-Glutamicol in the Lichen *Cladonia arbuscula* subsp. *squarrosa* under Seasonal Changes and Elevated Exposure to UV-B or PAR Irradiation

**DOI:** 10.3390/metabo12070632

**Published:** 2022-07-10

**Authors:** Ewelina Chrapusta-Srebrny, Jan Bialczyk, Kornelia Duchnik, Beata Bober

**Affiliations:** 1 Department of Plant Physiology and Development, Faculty of Biochemistry, Biophysics and Biotechnology, Jagiellonian University, Gronostajowa 7, 30-387 Krakow, Poland; j.bialczyk@uj.edu.pl (J.B.); kornelia.zabaglo@uj.edu.pl (K.D.); boberb@uek.krakow.pl (B.B.); 2 Department of Microbiology, Institute of Quality Sciences and Product Management, Cracow University of Economics, Rakowicka 27, 31-510 Krakow, Poland

**Keywords:** Myc-Glu(OH), mycosporine-like amino acid, PAR exposure, stress conditions, UV protection, UV-B exposure

## Abstract

*Cladonia arbuscula* in its environmental niches is regularly affected by daily and annual variations in solar radiation. Mycosporine-glutamicol, Myc-Glu(OH), which it synthesizes, may act as a significant cellular UV-protector. Therefore, we studied this compound concentration in lichen thalli concerning seasonal changes and increased exposure to UV-B and photosynthetically active radiation (PAR) with/without simultaneous CO_2_ deprivation. Myc-Glu(OH) occurred year-round and exhibited a strong seasonality. The most crucial role in the control of its synthesis played UV-B radiation, although its high concentration was also found after PAR irradiation at 1000 µmol m^−2^ s^−1^. As PAR intensity increased to 2000 µmol m^−2^ s^−1^, the rate of Myc-Glu(OH) synthesis slowed down. In turn, under dark/PAR irradiation with simultaneous deprivation of CO_2_ in the atmosphere surrounding *C. arbuscula* and during darkness with continuous access to atmospheric CO_2_, its production was insignificant. Obtained data confirmed that Myc-Glu(OH) plays an important role in protecting *C. arbuscula* from UV damage and favours its adaptation to environmental stress in its natural habitat. They also suggest that its synthesis is a synergism of multiple factors. Consequently, further studies should focus on their evaluation and the identification of a lichen partner actively involved in Myc-Glu(OH) biogenesis.

## 1. Introduction

Lichen *Cladonia arbuscula* is permanently subjected in its natural habitat to strong solar irradiation including photosynthetically active radiation (PAR) and ultraviolet radiation (UV). The excessive doses of UV-B (280–315 nm) can directly induce conformational modifications in DNA via the formation of cyclobutane-pyrimidine dimers (CPDs) and 6-4 pyrimidine-pyrimidine (6-4-PP) photoproducts leading to genetic mutations, whereas UV-A (315–400 nm) can trigger the production of reactive oxygen species (ROS) that promote cellular damage of nucleic acids, proteins and lipids [1,2].

The key feature of many taxonomically different organisms to successful colonization of sunlit niches is to develop a variety of strategies to prevent or counteract the deleterious effects of UV irradiation. Besides the DNA reparative mechanisms, they show a synthesis of antioxidants, detoxifying enzymes, and/or UV-screening compounds such as mycosporine-like amino acids (MAAs) [3,4,5,6,7,8,9,10]. MAAs are low-molecular metabolites characterized by a cyclohexenone or cyclohexenimine chromophore conjugated with a nitrogen moiety and the capability of UV absorption in the harmful range from 309 to 362 nm [1,9,11,12,13].

**Figure 1:**
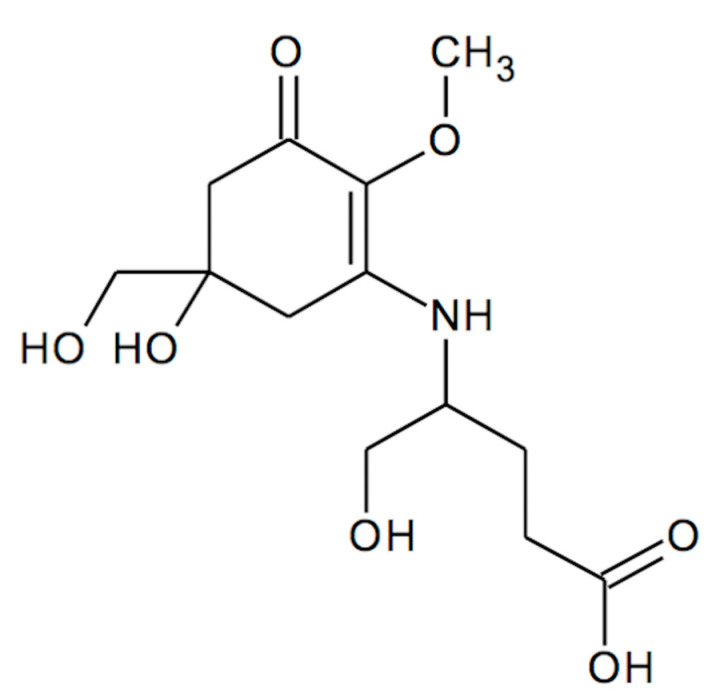
Myc-Glu(OH) chemical structure.

Our previous research revealed in *C. arbuscula* thalli the presence of MAA compound, which was identified as mycosporine-glutamicol, Myc-Glu(OH) [14] (Figure 1). Based on the obtained results, we hypothesized that this compound may act as the significant cellular UV-protector and its concentration might be a function of irradiation dose. Therefore, in the present study, we have investigated the kinetics of its accumulation (a) throughout the whole calendar year to verify its possible seasonal variation in natural community and (b) under laboratory conditions simulating elevated exposure to UV-B or PAR irradiation, with or without simultaneous deprivation of CO_2,_ to understand the mechanisms of this adaptive strategy.

## 2. Materials and Methods

### 2.1. Experimental Organism

The experimental organism *Cladonia arbuscula* (Wallr.) Flot subsp. *squarrosa* (Wallr.) Ruoss was harvested in April 2014 in Sobkow-Wolica (50°44′17.4″ N, 20°25′56.1″ E), Poland, and identified based on morphological characteristics with the help of standard taxonomic keys [15,16]. A voucher specimen has been kept for future references in the herbarium of the Department of Plant Physiology and Development, Jagiellonian University, Krakow, Poland.

### 2.2. Experimental Conditions

The naturally wetted *C. arbuscula* thalli (15 g fresh weight each) were placed in small beakers and then in glass vessels ¼ filled with water. Immediately afterwards lichen-forming fungi were subjected to the effect of a specified factor:

- PAR (λ = 400–700 nm) at the intensity of 1000, 1500, and 2000 µmol m^−2^ s^−1^ (two 1000 W Tungsram lamps, The Netherlands, 12 h light: 12 h dark) or darkness (24 h) with continuous access to atmospheric CO_2_ levels;
- UV-B radiation (λ = 290–315 nm, λ_max_ = 310 nm) of 5 µmol m^−2^ s^−1^ (two fluorescence tubes, Philips TL 40W/12, Germany, screened with a 0.13 mm thick cellulose acetate filter paper to remove all radiation below 290 nm);
- PAR of 1000 µmol m^−2^ s^−1^ or darkness with simultaneous deprivation of CO_2_ in the atmosphere surrounding lichen-forming fungus, which was achieved by replacing water in a glass vessel with a 5% Ba(OH)_2_ solution.

Under the above conditions, *C. arbuscula* was cultivated over 49 days in the growth chamber at 21 ± 1 °C and humidity of 80%. Irradiation intensity was measured inside the vessels using a Photometer-Radiometer (HD 2102.1/2102.2, Delta OHM, Selvazzano Dentro, Italy) with LP471UVB and LP471PAR probes. Samples for analysis were collected at 0, 7th, 14th, 28th, and 49th day of treatment. The material was divided into the upper (tip) and lower (stem) parts of podetia.

### 2.3. Kinetics of the Changes in Myc-Glu(OH) Concentration 

To evaluate the concentration of Myc-Glu(OH) in *C. arbuscula*, during the changing seasons, its thallus was collected cyclically 4 times a month at weekly intervals in 2014. 

### 2.4. Sample Preparation

All samples of the lichen-forming fungus were air-dried, crushed, weighed in equal portions, and immersed in 25% methanol (MeOH) (*v*/*v*) for 3 h at 45 ± 1 °C according to the procedure described by Tartarotti and Sommaruga [17]. The supernatants were filtrated through Whatman^TM^ glass filters GF/C (USA) ∅ 0.45 μm, centrifuged (10,000× *g*, 5 min), and evaporated to dryness under reduced pressure at 21 ± 1 °C. Pellets were re-suspended in 100% MeOH, and aliquots were re-centrifuged and re-evaporated. The dry residues were re-dissolved in Milli-Q water followed by the addition of amounts of chloroform with gentle vortexing. After centrifugation, the uppermost water phases were filtered through the Durapore syringe filters (Durapore, Germany) ∅ 0.22 μm and kept at −20 ± 1 °C for high-pressure liquid chromatography (HPLC) analysis.

### 2.5. Analytical Determination

Chromatographic analyses were conducted using a Waters (Waters, Milford, MA, USA) apparatus consisting of a 600E gradient pump, 717 plus autosampler, 996 photodiode detector, Millenium^32^ SS Software, and a Jetstream 2 plus column oven. Qualitative and quantitative identification of Myc-Glu(OH) was performed on an analytical column Waters Atlantis^®^ dC18 (3.9 × 100 mm; 3 μm) with guard column C_18_ thermostated at 35 ± 1 °C by using a mobile phase consisting of water/acetonitrile (ACN); both were acidified with 0.05% trifluoroacetic acid (TFA, *v*:*v*). A linear gradient was set from 100% to 77% water in 6 min at a flow rate of 1 mL min^−1^. The Myc-Glu(OH) was identified by comparing the UV spectra determined for prepared standard and quantified by absorbance at λ_max_ = 309 nm using the calibration curve.

### 2.6. Reagents and Chemicals

All the chemicals were analytical or HPLC grade and were purchased from Sigma-Aldrich (USA). Milli-Q water was obtained using an Elga (Elga LabWater, High Wycombe, UK) Maxima LS Ultra-Pure Water Purifier.

### 2.7. Statistical Analysis

The results were presented as means ± standard deviation (S.D.) of five independent replicates. Data were analysed using analysis of variance (*ANOVA*) in Statistica 10 software (StatSoft, Tulsa, OK, USA). Differences were considered significant at *p* < 0.05 [18].

## 3. Results and Discussion

### 3.1. Kinetics of Myc-Glu(OH) Concentration in C. arbuscula Thalli

**Figure 2:**
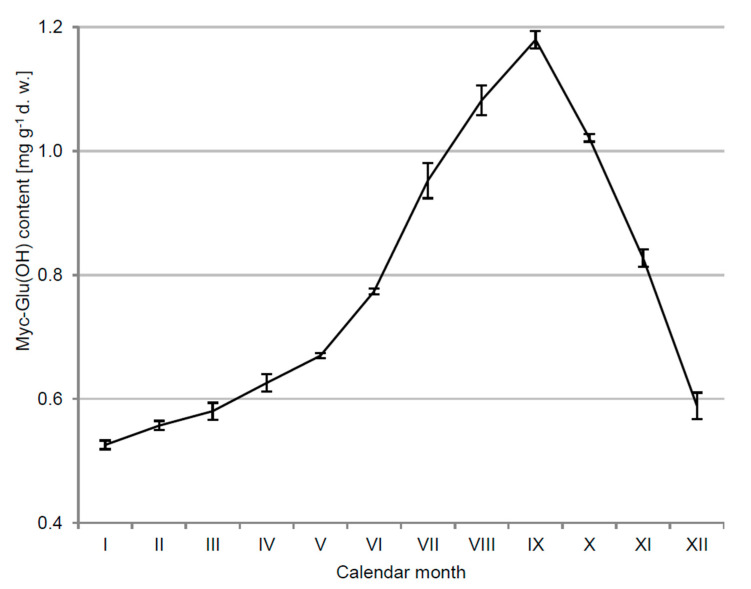
The concentration of Myc-Glu(OH) in *C. arbuscula* thalli throughout 2014 calendar year; *n* = 5 ± S.D., d.w.—dry weight.

*C. arbuscula* in its ecological niches is regularly affected by daily and annual variations in solar radiation. For lichen-forming fungi, numerous studies show a strong correlation between the environmental factors and the intensity of secondary metabolites production [19]. This connection also refers to the biosynthesis of Myc-Glu(OH). Its concentration in *C. arbuscula* thalli exhibited a clear seasonality, ranging from 0.526 ± 0.007 mg g^−1^ of dry weight (d.w.) in January to 1.179 ± 0.014 mg g^−1^ d.w. in September 2014 (>2-fold increase) (Figure 2). The elevational trend in Myc-Glu(OH) accumulation over the summer was correlated with the greater intensity of solar energy reaching the Earth’s surface and clearly confirmed its relevance in acclimatization of the lichen-forming fungus to such conditions. In turn, a decrease in the concentration of this highly polar and water-soluble compound in October was probably related to its leaching out from the thallus by rainwater. Previously, potential seasonal fluctuations in the quantitative and qualitative composition of MAAs have been verified only for freshwater and marine species [20,21,22,23,24,25,26]. However, they did not demonstrate one common pattern for all tested organisms. A similar annual variation to the Myc-Glu(OH) occurrence was reported for MAAs in the phytoplankton cells and copepod *Cyclops abyssorum tatricus* body from a transparent alpine lake [24]. Both organisms showed, respectively, a 3.6 and 3-fold higher level of MAAs synthesis in the summer compared to winter months. The MAAs concentration in phytoplankton collected from the English Channel was also closely correlated with seasonal changes in solar radiation intensity [22], whereas such a pattern was not observed for the Antarctic sea urchin *Sterechinus neumayeri* [20]. Comparable annual variability to the Myc-Glu(OH) accumulation was also revealed for other photoprotective compounds. The intensity of melanin production by the subarctic *Daphnia* spp. significantly increased after the ice-break [27], and the concentration of parietin in the lichen *Xanthoria parietina* exhibited a rapid spring growth and an autumn decline [28].

Generally, MAAs concentration does not exceed 1% of dry weight [1]. Similar values to the Myc-Glu(OH) occurrence in *C. arbuscula* thalli were determined for mycosporine-glycine in the lichen *Lichina pygmae*; the concentration of this compound was equal to 1.11 ± 0.23 mg g^−1^ d.w. [29]. A significantly higher mycosporine-glycine concentration, from 0.1 to 0.8% of d.w., characterized lichens belonging to the *Collema*, *Gonohymenia*, and *Peltula* genera [30].

### 3.2. Regulation of Myc-Glu(OH) Accumulation

**Figure 3:**
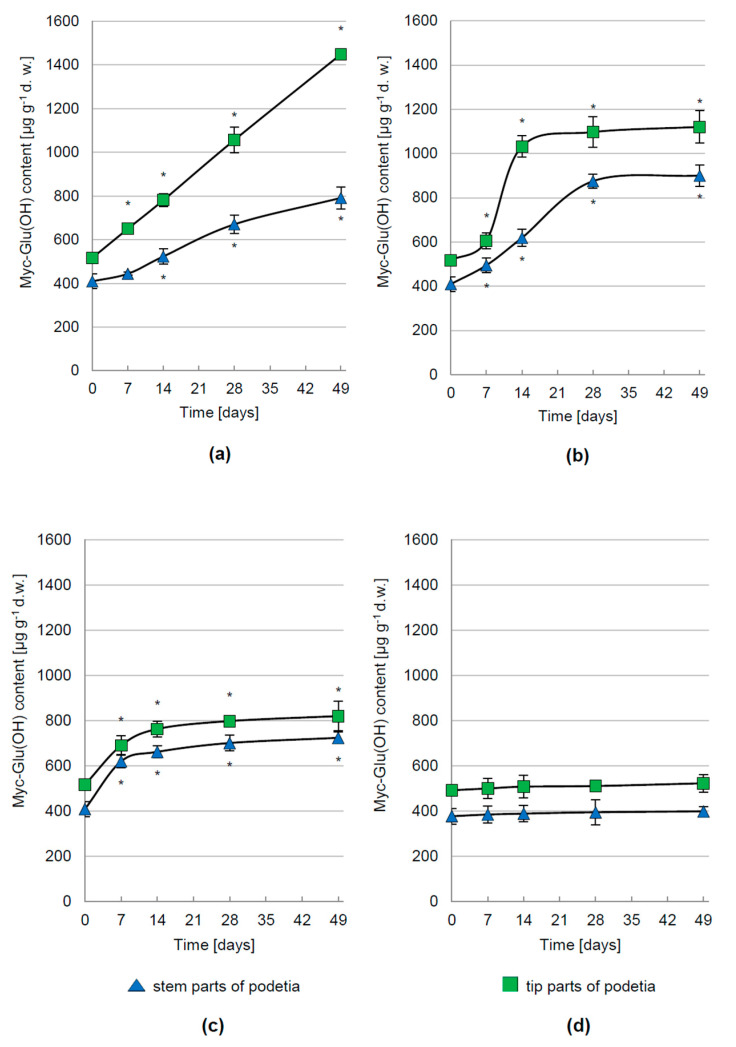
The concentration of Myc-Glu(OH) in *C. arbuscula* thalli exposed to PAR at intensity of: (**a**) 1000 μmol m^−2^ s^−1^; (**b**) 1500 μmol m^−2^ s^−1^; and (**c**) 2000 μmol m^−2^ s^−1^ and (**d**) darkness conditions; *n* = 5 ± S.D.; * *p* < 0.05, d.w.—dry weight.

In vivo experiments showed that the production of Myc-Glu(OH) is controlled not only by different intensity but also the spectral composition of solar rays. PAR was one of the important factors determining the synthesis level of Myc-Glu(OH) in *C. arbuscula* thalli, but the kinetics of this process depended on the applied intensity. The greatest increase in its concentration by 180% (1448 µg g^−1^ d.w.) and 93% (791 µg g^−1^ d.w.) in the tip and stem parts of podetia, respectively, was determined after 49 days of treatment with 1000 µmol m^−2^ s^−1^ (Figure 3a). Along with the elevation of PAR radiation up to 1500 µmol m^−2^ s^−1^, the rate of Myc-Glu(OH) production slowed down and at the end of the experiment its accumulation enhanced by 117% (1120 µg g^−1^ d.w.) in the tip and 120% (900 µg g^−1^ d.w.) in the stem parts of podetia compared to their initial values (Figure 3b). In turn, the highest tested PAR dose of 2000 µmol m^−2^ s^−1^ stimulated the Myc-Glu(OH) production only at the beginning of the cultivation, followed by the stabilization period (Figure 3c). Finally, its concentration was equal to 820 µg g^−1^ d.w. in the tip (an increase of 77%) and 724 µg g^−1^ d.w. in the stem parts of podetia (an increase of 59%). The lower level of metabolite synthesis under higher light intensities may be the effect of photosynthetic apparatus damage. 

Similar hypersensitivity was documented in hydrated thalli of several lichen species [31]. The elevated production of MAAs and changes in their composition under PAR irradiation was also demonstrated for the dinoflagellate *Alexandrium excavatum* moving from low (20 μE m^−2^ s^−1^) to 10 times greater light intensities [32]. In turn, a 14-fold higher concentration of MAAs was reported for the dinoflagellate *Gymnodinium sanguineum* cultured in the cold white fluorescent light of approx. 350 μmol m^−2^ s^−1^ compared to those treated with approx. 70 μmol m^−2^ s^−1^ [33]. MAAs accumulation was also induced by different spectral compositions (white, blue, green, yellow, and red light). Korbee et al. described the beneficial role of blue light on porphyra-334, palythine, and asterine-330 synthesis in the red alga *Porphyra leucosticta*, whereas white, green, yellow, and red light favoured the production of shinorine [34].

**Figure 4:**
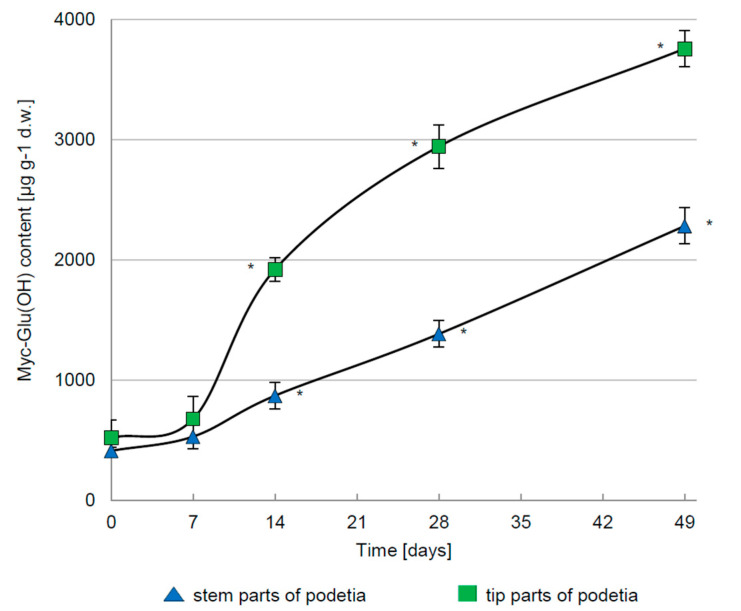
The concentration of Myc-Glu(OH) in *C. arbuscula* thalli exposed to UV-B irradiation at intensity of 5 μmol m^−2^ s^−1^; *n* = 5 ± S.D.; * *p* < 0.05, d.w.—dry weight.

Despite the unquestionable importance of PAR in the control of the Myc-Glu(OH) synthesis in *C. arbuscula* thalli, the most crucial role in this process was played by UV-B radiation. Under 7-weeks exposition to 5 μmol m^−2^ s^−1^ the concentration of this metabolite was significantly elevated by 624% (3758 μg g^−1^ d.w.) and 457% (2282 μg g^−1^ d.w.) in the tip and stem parts of the podetia, respectively (Figure 4). The positive influence of UV-B on MAAs accumulation was also observed in the polar algae *Phaeocystis antarctica* [35], dinoflagellates *Alexandrium tamarense* and *Heterocapsa triquetra* [36], and nitrogen-fixing cyanobacteria [6]. Compared to other wavelengths, UV-B exerted a more pronounced influence on MAAs synthesis in cyanobacteria [37]. *Arthrospira* sp. CU2556 and *Gloeocapsa* sp. treated simultaneously with PAR + UV-A + UV-B exhibited an effective increase in the production of mycosporine-glycine [38], shinorine, and M-307 [39], rather than irradiated with PAR or PAR + UV-A. The induction of palythine and mycosporine-glycine synthesis by the marine red alga *Devaleraea ramentacea* was also revealed after exposure to PAR in combination with UV-A and UV-B [40]. In contrast, the concentration of usnic acid (depsidone) and atranorin (depside), synthesized in addition to Myc-Glu(OH) by studied lichen-forming fungus, was affected mainly by UV-A [41]. Similar wavelength ranges stimulated the production of MAAs by Arctic diatoms [35,42,43]. However, opposite to the above examples, for some species such correlations have not been recorded at all [44].

De novo MAAs synthesis is a highly energetic and costly process; nevertheless, these costs are disproportionate to UV-induced damage [23,45]. Their presence in a cell can effectively prevent 3 out of 10 photons from hitting cytoplasmic targets [46]. Thus, an increase in the production rate of MAAs, including Myc-Glu(OH), due to excessive exposure to high doses of UV effectively reduces metabolic costs until the ecological benefits of their presence will be achieved [47].

Distribution of Myc-Glu(OH) within the *C. arbuscula* thalli irradiated with PAR or UV-B was not homogeneous. The difference in its concentration probably reflects (1) various metabolic activity of the enzymes controlling its synthesis in the upper, younger parts and in the lower, older parts of podetia [48]; (2) evolutionary adaptation of this lichen-forming fungus to UV protection of upper parts containing reproductive structures such as pycnidia; (3) a different degree of the exposure of the particular thalli parts to UV. The tip parts of podetia form branches that naturally shade the lower parts [49]; therefore, the enhanced synthesis of Myc-Glu(OH) by the latter may not be necessary. Similar dependence, as for the tested Myc-Glu(OH), was demonstrated for usnic acid in *Flavocetraria nivalis* [50] and phenolic compounds that effectively block the amount of UV-B penetrating into *Cladonia mitis* thalli [49].

**Figure 5:**
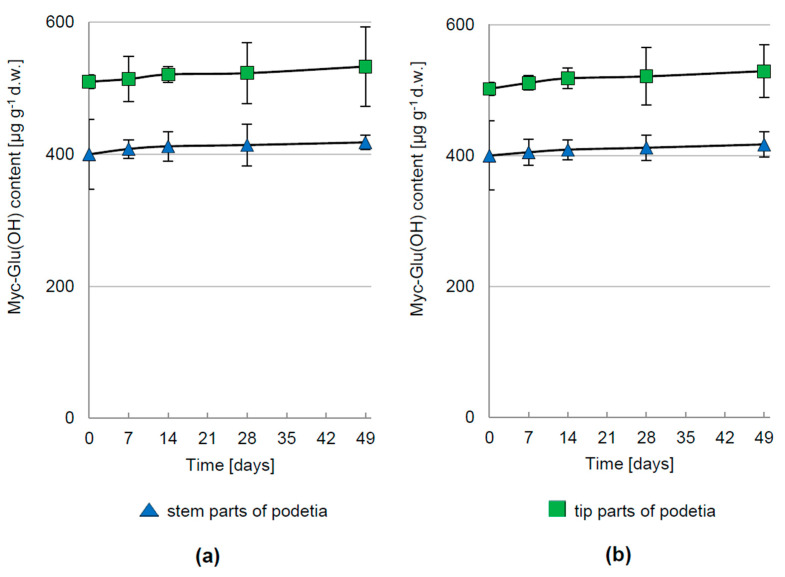
The concentration of Myc-Glu(OH) in *C. arbuscula* thalli under conditions of CO_2_ deprivation in the ambient atmosphere with simultaneous exposure to: (**a**) PAR at intensity of 1000 μmol m^−2^ s^−1^; (**b**) darkness; *n* = 5 ± S.D.; d.w.—dry weight.

Under dark or PAR irradiation (1000 μmol m^−2^ s^−1^) conditions with simultaneous deprivation of CO_2_ in the atmosphere surrounding *C. arbuscula*, the Myc-Glu(OH) concentration remained constant over the whole cultivation period (Figure 5a,b). Its comparatively insignificant production was also demonstrated during darkness with continuous access to atmospheric CO_2_ levels (Figure 3d). 

Therefore, it is suggested that the synthesis of the analysed metabolite is dependent on the availability of light and CO_2_. Based on these results, we assume that the carbon compounds to Myc-Glu(OH) production are formed during the green algal photosynthesis rather than the fungal dark carboxylation. On the other hand, Myc-Glu(OH) possesses a carbonyl group characteristic for fungal mycosporines, which may suggest a significant contribution of mycobiont in its biogenesis. This hypothesis is also supported by the fact that Myc-Glu(OH) has been detected so far in the microcolonial fungi and sclerotia of the deuteromycete *Botrytis cinerea* [51,52,53,54]. Furthermore, the literature data clearly show that many of the UV-absorbing compounds, such as polyphenolics, anthraquinone pigments, or usnic acid are produced by mycobionts [30]. Nevertheless, both lichen partners may be involved in the biogenesis of Myc-Glu(OH); especially because the fungi and green algae, as independent organisms, are known for MAAs synthesis [55]. Only a few studies have shown that the production of MAAs has been clearly defined and attributed to one particular component. Torres et al. determined that the collemine A is produced by mycobiont of the *Collema cristatum* [56]. In addition, Roullier et al. revealed that cephalodia of the *Stereocaulon philippinense*, composed of cyanobacterial cells, contained MAA with λ_max_ at 340 nm instead of the compound present in the whole thalli with λ_max_ = 310 nm [57]. They assumed that one partner could synthesize MAA with λ_max_ = 310 nm, and the other structurally modified it to the form with λ_max_ = 340 nm. Although the parallel, uncorrelated and independent biogenesis of those two compounds in different parts of the thalli cannot be excluded. Some cyanobacteria, due to the ability to atmospheric nitrogen fixation, appear to be involved in the production of MAAs in cyanolichens.

## 4. Conclusions

The annual pattern of Myc-Glu(OH) content exhibited a clear seasonality with a more than 2-fold increase in concentration during elevated exposure to solar radiation in late summer. Its biosynthesis is regulated by UV-B and PAR, the effect of the latter being markedly weaker. These findings are consistent with the idea that this compound provides adaptation of *C. arbuscula* to relatively high radiation levels and irradiance changes in the natural habitat. Nevertheless, the enhanced biosynthesis of Myc-Glu(OH) is unquestionably a synergistic effect of many factors rather than individual PAR or UV-B influence as demonstrated by experiments with/without the presence of CO_2_ in the atmosphere surrounding lichen. Consequently, further research should focus on understanding these factors. Given that the accumulation of Myc-Glu(OH) in the *C. arbuscula* thalli is a benefit of this symbiosis, it would be useful to concentrate future studies on the identification of lichen partners actively involved in its biogenesis.

## Data Availability

The data presented in this study are available on request from the corresponding author. The data are not publicly available due to the fact that they were provided in the manuscript.
